# Omics-based integrated analysis identified IKZF2 as a biomarker associated with lupus nephritis

**DOI:** 10.1038/s41598-022-13336-5

**Published:** 2022-06-10

**Authors:** Mi Zhou, Yuening Kang, Jun Li, Rongxiu Li, Liangjing Lu

**Affiliations:** 1 Department of Rheumatology, School of Medicine, Renji Hospital, Shanghai Jiaotong University, Shanghai, 200001 China; 2 State Key Laboratory of Microbial Metabolism and School of Life Sciences and Biotechnology, Shanghai Jiao Tong University, 800 Dong Chuan Road, Shanghai, 200240 China

**Keywords:** Immunological disorders, Kidney diseases, Rheumatic diseases, Computational biology and bioinformatics, Immunology, Biomarkers, Diseases, Molecular medicine, Nephrology, Pathogenesis, Rheumatology, Risk factors

## Abstract

Lupus nephritis (LN) is a crucial complication of systemic lupus erythematosus (SLE). IKZF2 was identified as a lupus susceptibility locus, while its exact molecular function in LN is unknown. We aimed to explore the relationship between IKZF2 and LN based on multi-omics data. In our study, we carried out a meta-analysis of publicly available data, including not only tubulointerstitium, but also glomerulus tissue samples from LN patients and controls. Based on the common differentially expressed genes (co-DEGs) and previous researches, we selected IKZF2 for further analysis. Then, we analyzed potential molecular mechanisms of co-DEGs and IKZF2 in LN. To explore the possible targets of IKZF2, protein–protein interaction network (PPI) network and ceRNA network of IKZF2 were also constructed. Moreover, we performed immune infiltration analysis and evaluated clinical value of IKZF2. A total of 26 co-DEGs were observed in the integration of the above DEGs coming from the four sets of data, of which IKZF2 was selected for further analysis. Functional enrichment analysis from IKZF2 and related PPI network confirmed the tight relationship between IKZF2 and the immune reaction. Moreover, immune filtration analysis revealed the significant correlation between IKZF2 and naïve B cell, NK cell activation, NK cell rest and other immune cells. Receiver operating characteristic (ROC) analysis showed that the areas under the ROC curves were 0.721, 0.80, 0.682, and 0.859 for IKZF2 in four datasets, which demonstrated the clinical value of IKZF2. Our study revealed that IKZF2 may play an essential role in the molecular function and development of LN, and might be a potential biomarker for distinguishing LN patients and healthy ones.

## Introduction

Systemic lupus erythematosus (SLE) is a multifactorial and multisystem autoimmune disease that commonly affects the kidneys^1^. LN is a major driver of mortality of SLE patients^2^, and up to 20% of SLE patients who have been afflicted by LN will finally develop end-stage kidney disease (ESKD) within the first decade of the disease course^3^. However, on the one hand, the clinical detection of early disease can be challenging because patients often lack overt signs of kidney disease^4^, and the conventional parameters such as proteinuria and serum creatinine are insensitive and non-specific^5^. On the other hand, current therapies are not sufficiently efficacious to control LN and not all patients show adequate treatment responses^6^. Thus, it is critical and necessary to search and identify novel candidate biomarkers or therapeutic targets for LN.

Big data genomic assays have advanced our understanding of the molecular heterogeneity of SLE^7^. Genome-wide association studies (GWASs) in SLE have revealed more than 80 susceptibility loci^8–10^. Nevertheless, the identified risk variants could only explain a small portion of heritability of SLE^11^. Besides, gene expression, as an intermediate phenotype between DNA and disease phenotypic variation, can provide us with a new perspective to our understanding of the molecular mechanisms leading to SLE^12^. IKZF2, also known as HELIOS, was initially thought to be restricted to Treg cells^13,14^. In Treg cells, it was claimed to control IL2 expression by promote binding of Foxp3 to the IL2 promoter^15^. It is expressed in other immune cells, epithelial tissues of the gut, the respiratory tract, and the tubules of the kidney^13^ etc. Interestingly, it was also identified as a lupus susceptibility locus by GWASs^9,16^, and a recent research found it was enriched to regulating the related gene for T cells, B cells, and peripheral blood cells (PBCs) in SLE^17^. However, its exact molecular function in LN is still unknown^18^.

In our study, we carried out a meta-analysis of publicly available data including not only tubulointerstitium but also glomerulus tissue samples from LN patients and pre-transplant healthy living donors (LD), and we identified common differentially expressed genes (co-DEGs) for each dataset. Among 26 co-DEGs, we selected IKZF2 for further analysis based on the GWASs^8–10^ and previous researches^9,13–18^. Then, we analyzed potential molecular mechanisms of co-DEGs and IKZF2 in LN. Protein–protein interaction network (PPI) network and ceRNA network of IKZF2 were also constructed to explore the possible targets of IKZF2. Moreover, we performed immune infiltration analysis and evaluated clinical value of IKZF2. Over all, this is the first study applying multiple datasets to understand the molecular mechanism of IKZF2 in LN in detail.

## Materials and methods

### Data collection

We mined the Gene Expression Omnibus (GEO) database^19^ to find publicly available gene expression datasets for LN including both tubulointerstitium or glomerulus tissue samples. The datasets of GSE32591^20^ and GSE113342^21^ were selected and downloaded by the R package GEOquery^22^. Each dataset was divided into two groups of tubulointerstitium (TUB) or glomerulus (GLOM) for subsequent analysis. For details of the LN cases and LD included in the two gene expression profiles, see Table 1.

**Table 1:** Details of the LN cases and LD included in the two gene expression profiles.

ID	GLOM (LN)	GLOM (LD)	TUB (LN)	*TUB* (LD)
GSE32591	32	14	32	15
GSE113342	28	6	28	10

### Common differentially expressed genes (co-DEGs)

Differentially expressed genes (DEGs) between LN cases and LD were identified by R package Limma^23^ in the two gene expression profiles for the tubulointerstitium and glomerulus tissue type, respectively. The criteria of DEGs was defined with adjust *P*-value < 0.05, and up/down-regulated DEGs was defined with adjust *p*-value < 0.05 and |logFC|> 1. Then, the co-DEGs was the intersection of the four gene sets. Thus,

The DEGs of each dataset were also integrated with R package “RobustRankAggreg”^24^, one meta-analysis method, in order to make the result robust. In all, the results of DEGs were visualized by the R package ggplot2^25^, pheatmap^26^, and VennDiagram^27^.

### Functional enrichment analysis of co-DEGs

Gene Ontology (GO) analysis is commonly conducted for large-scale functional enrichment study^28^. Kyoto Encyclopedia of Genes and Genomes (KEGG, www.kegg.jp/kegg/kegg1.html) pathway is a database regarding diseases, genomes, biological pathways, chemical and drugs substances^29^. Disease Ontology (DO)^30^ annotates genes based on human diseases, helping to transform molecular discovery from high-throughput data into clinical relevance. We used the R package cluster Profiler^31^ to perform GO and KEGG analysis, and used the R package DOSE^32^ to conduct DO annotation. *P*-value < 0.05 was considered to be statistically significant for enrichment analysis.

### IKZF2-associated protein‑protein interaction (PPI) network

STRING^33^ is a public data source of known and predicted protein–protein interactions (PPI), from which we extracted the PPI of IKZF2-associated genes and used cytoscape^34^ to perform visualization. Then, we applied cytoscape plugin Molecular Complex Detection (MCODE)^35^ to identified highly interconnected clusters in the IKZF2-associated PPI network, and annotated GO terms of the specific cluster by ClueGO^36^ plug-in. Besides, the Database for Annotation, Visualization, and Integrate Discovery (DAVID)^37^and Gene Set Enrichment Analysis (GSEA)^38^ were also used to understand biological significance underlying the IKZF2-associated PPI network.

### Construction of ceRNA network

To further analyze the potential targets and biological function of IKZF2 in LN, we constructed a competing endogenous RNA (ceRNA)^39^ network related to IKZF2 including the mRNA, miRNA and lncRNA. The prediction of IKZF2-related miRNA targets was obtained from the starBase (http://starbase.sysu.edu.cn/index.php)^40^, miRDB (http://www.mirdb.org/)^41^, Targetscan^42^ (http://www.targetscan.org/vert_72/), TarBase.v.8 (http://carolina.imis.athena-innovation.gr/)^43^. miRNA-related lncRNA targets were predicted by miRNet (https://www.mirnet.ca)^44^. Based on miRNA-mRNA and miRNA-LncRNA, a ceRNA network was established and visualized using Cytoscape.

### Immune infiltration analysis

For the co-DEGs between LN cases and LD of the tubulointerstitium or glomerulus group in the two datasets, immune infiltration analysis was performed using CIBERSORT^45^, which was based linear support vector regression to deconvolute relative abundance of 22 infiltrated immune cells. Principal components analysis (PCA) was applied to illustrate the differences in immune infiltrating cells between LN cases and LD, and the stromal/immune scores of each sample were shown by the R package pheatmap^26^. Besides, the correlation between the IKZF2 expression and the types of 22 immune cell was determined by Spearman’s correlation test^46^.

### Clinical characteristics correlation analysis and relative risk assessment

Spearman’s correlation test^46^ was adopted to explore the correlation between IKZF2 expression and clinical features. Receiver operating characteristic (ROC) analysis was applied to evaluate whether the expression of IKZF2 could distinguish LN tissues from normal tissues. R package pROC^47^ was used to calculate the area under the ROC curve and plot ROC curve.

## Results

### The identification of co-DEGs

**Figure 1:**
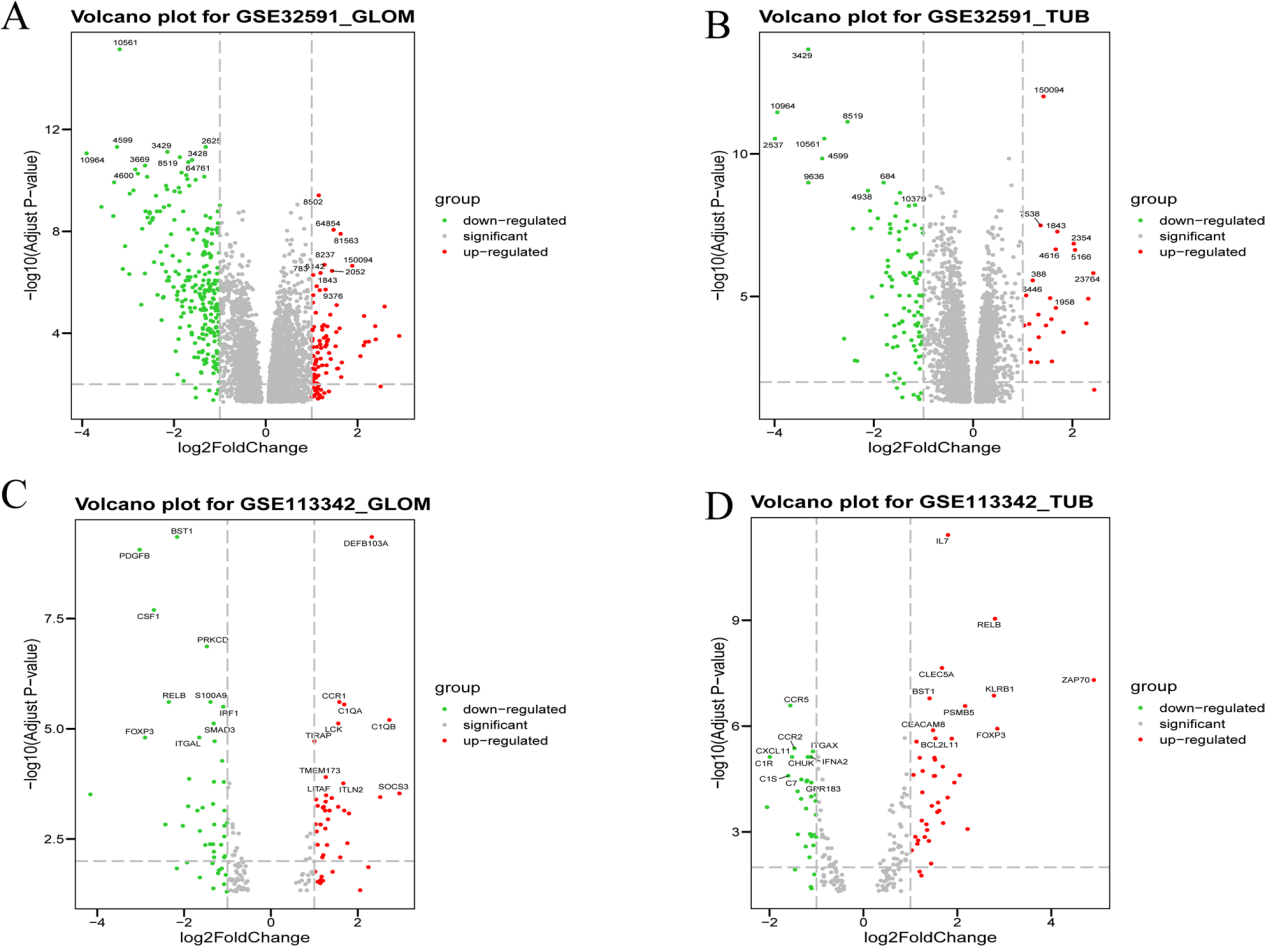
Volcano dots of differentially expressed genes (DEGs) from GSE32591 and GSE113342. Comparing with GSE113342, more DEGs were obtained from GSE32591. There were 4442 DEGs in GSE32591_GLOM group (**A**) and 5823 DEGs in GSE32591_TUB group (**B**), as well as 158 DEGs in GSE113342_GLOM (**C**) and 199 DEGs in GSE113342_TUB (**D**).

**Figure 2:**
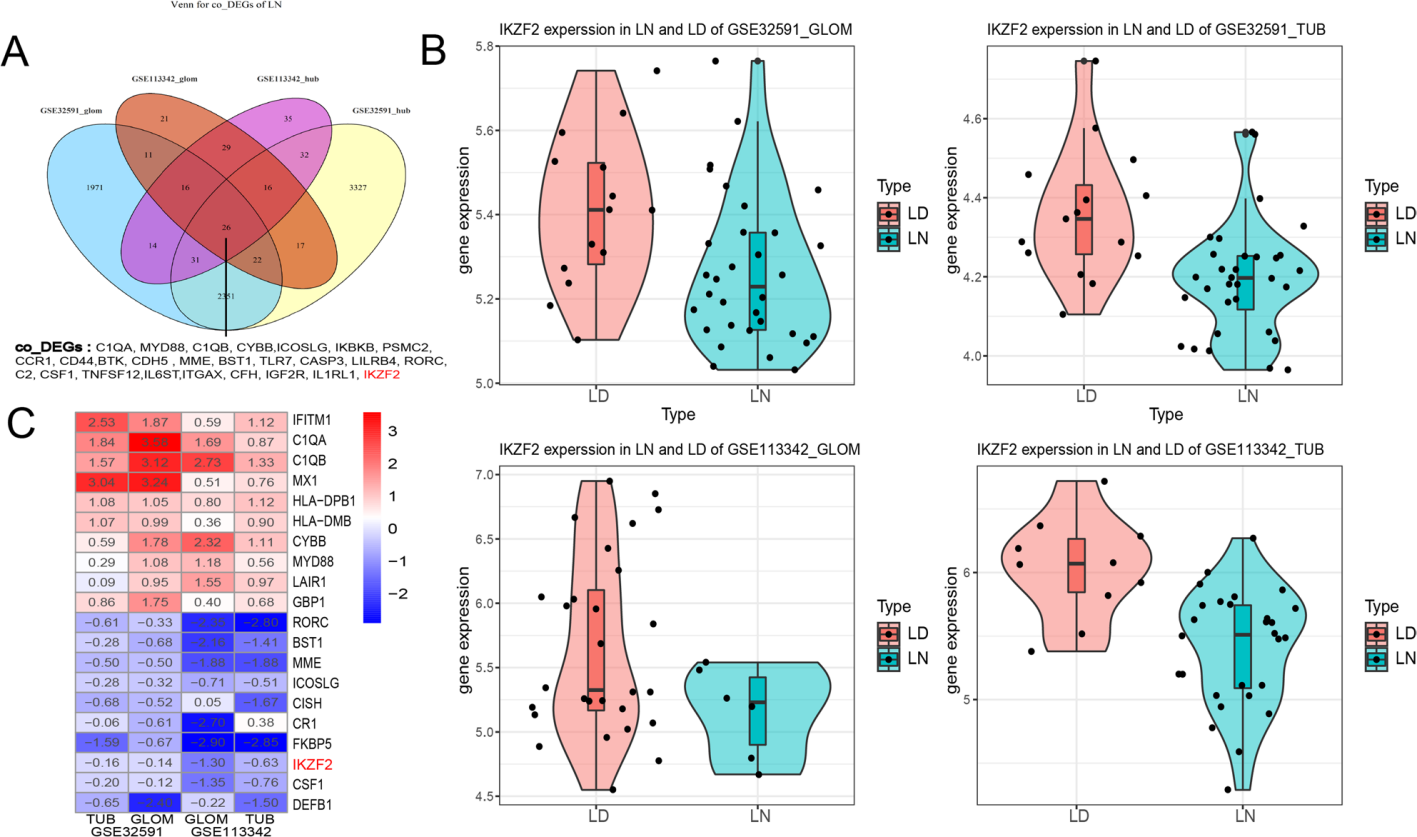
The expression level of co-DEGs and IKZF2 expression in each dataset. (**A**) The four colors involved in Venn diagram denoted the differentially expressed genes (DEGs) from GSE32591_TGLOM, GSE32591_TUB, GSE113342_ GLOM and GSE113342_TUB independently, and after integration of these DEGs, 26 co-DEGs were identified. (**B**) Comparison of IKZF2 expression value between LN/LD samples from every group of the two datasets. The results showed that IKZF2 was significantly down regulated in the LN samples when compared with healthy controls. (**C**) First 10 upregulated and downregulated DEGs of all datasets determined by “RobustRankAggreg”.

**Figure 3:**
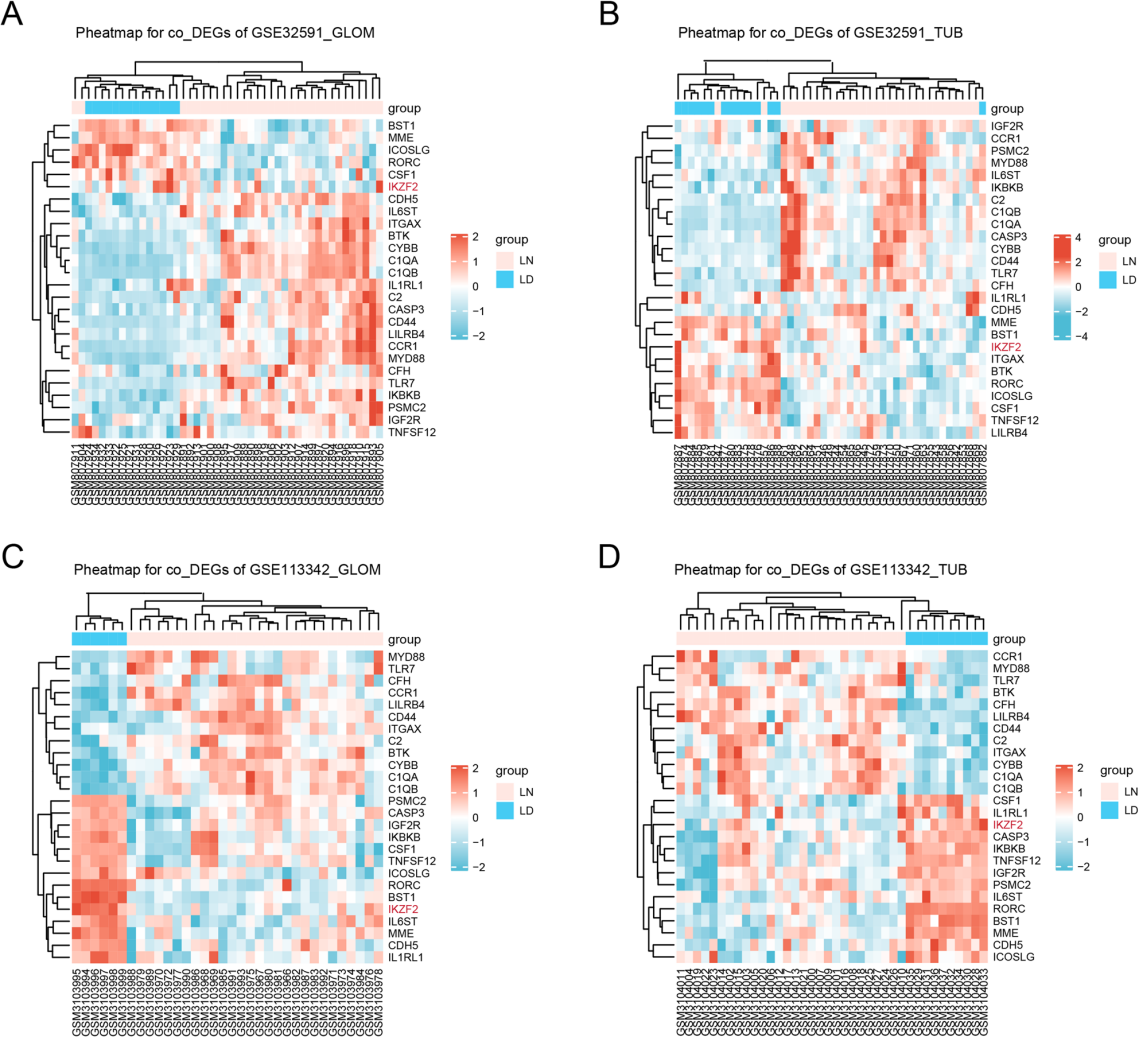
Identifying the correlation between co-DEGs and clinical diagnosis. Heat map of co-DEGs expression from the four sets of data. The abscissa depicted the id of patients and the ordinate denoted 26 co-DEGs. The above pink strip and the blue one represented LN patients and healthy controls respectively. The expression level of co-DEGs were shown in a descending order from red to blue (A-D). The results revealed that the 26 co-DEGs can easily distinguished LN samples from LD. Abbreviation, co-DEGs: common-differentially expressed genes.

To elucidate DEG between the sample from the LN cases and LD, the four samples from GSE32951 and GSE113342 datasets was analyzed using R package limma to identify the DEG and its related up/down- regulated genes according to the cutoff threshold (adjust *P*-value < 0.05, and |logFC|> 1 (Table 2 and Fig. 1A–D). A total of 26 co-DEGs were observed in the integration of the four sets of data (Fig. 2A). The color of heatmap represents each co-DEG expression in different sample (Fig. 3A–D). These results revealed that the 26 co-DEGs can easily distinguished LN samples from LD.

**Table 2:** Differentially expressed gene.

Group	DEGs	Up-genes	Down-genes
GSE32591- GLOM	4442	101	250
GSE32591- TUB	5823	25	104
GSE113342- GLOM	158	45	47
GSE113342- TUB	199	33	42

Based on the GWASs^8–10^ and previous researches^9,13–18^, we selected IKZF2 as the focus of the follow-up research. Compared with normal controls, the expression value of IKZF2 was significantly downregulated in the LN samples (Fig. 2B). Besides, IKZF2 was also shown in the first 10 downregulated DEGs of all datasets determined by “RobustRankAggreg” (Fig. 2C).

### Functional enrichment analysis of co-DEGs

**Figure 4:**
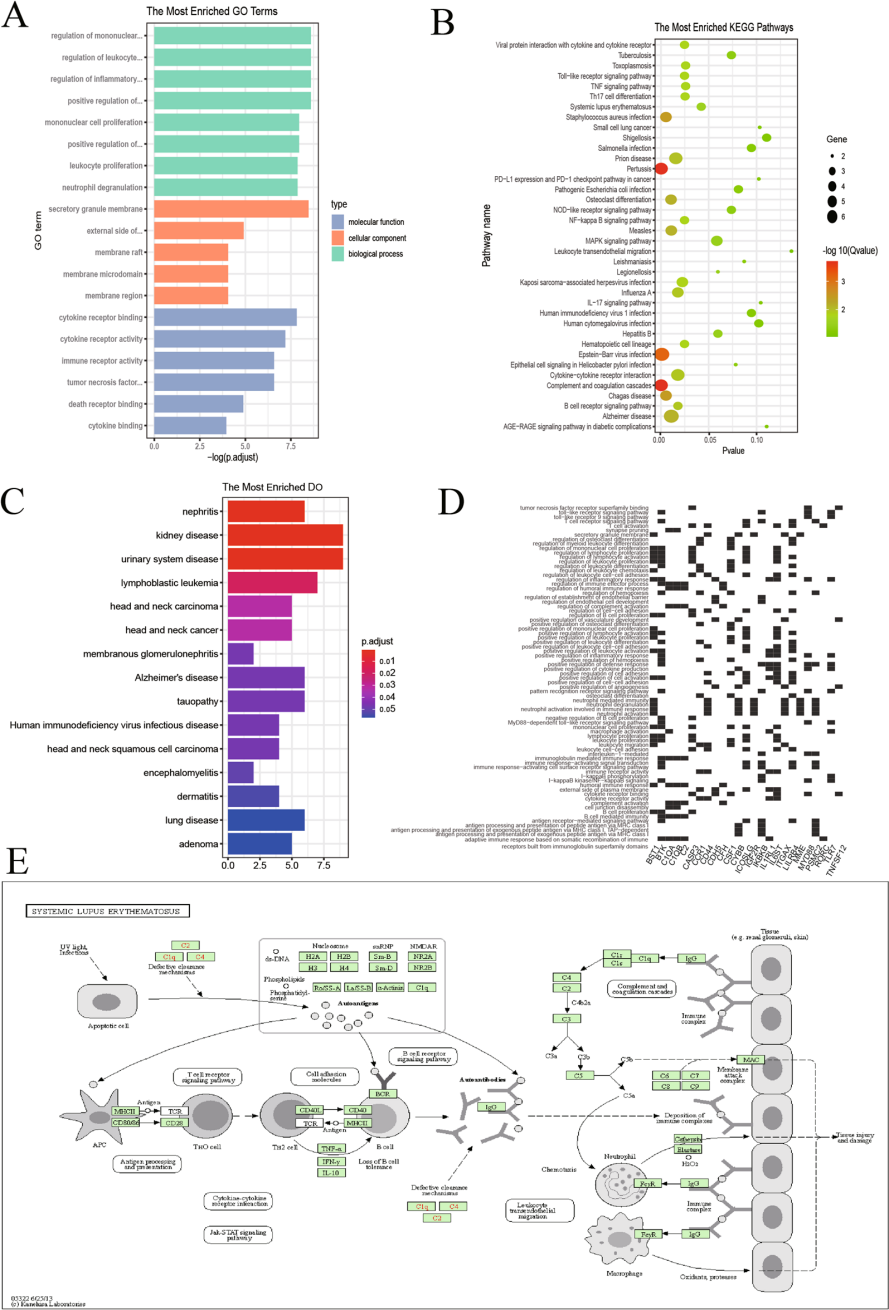
GO functional enrichment analysis, KEGG pathway enrichment analysis, and DO enrichment analysis. Three ways of enrichment analyses were performed upon the 26 co-DEGs. (**A**) GO analysis. The adjust p-value was calculated as x-axis and the GO terms was denoted as y-axis. Blue-purple strip, orange strip and range strip represented molecular function, cellular components and biological processes respectively. (**B**) The bubble diagram for the KEGG analysis results. Distinct size of dots represented the number of genes enriched in the corresponding pathway. (**C**) DO analysis. The x-axis represented 26 co-DEGs and the y-axis represented DO terms. (**D**) the co-DEGs in the enriched GO terms were particularly exhibited. (**E**) KEGG pathway associated with lupus nephritis.

In order to get further insight into the correlation between co-DEGs and biological processes, molecular functions, cellular components, biological pathways and specific diseases, functional enrichment was conducted. Firstly, GO analysis was conducted to reveal the biological characteristics of 26 co-DEGs. In the biological process category, these co-DEGs were mainly enriched in the mononuclear cell proliferation, leukocyte proliferation, regulation of inflammatory, etc. And from the point of molecular function, the hub mostly enriched GO terms included cytokine receptor binding, immune receptor activity, tumor necrosis factor and death receptor. Moreover, the cellular component category exhibited that co-DEGs mainly concentrated on the secretory granule membrane, membrane raft, membrane microdomain and membrane region (Fig. 4 A and Table 3).

**Table 3:** GO analysis of co-DEGs.

	Terms	*P* value
BP	Regulation of mononuclear cell proliferation	3.5E-07
	Regulation of leukocyte proliferation	4.99E-07
	Regulation of inflammatory response	5.8E-07
	Positive regulation of defense response	6.92E-07
	Mononuclear cell proliferation	1.7E-06
	Positive regulation of inflammatory response	1.98E-06
	Leukocyte proliferation	2.77E-06
	Neutrophil degranulation	3.28E-06
CC	Secretory granule membrane	2.02E-06
	External side of plasma membrane	0.000143
	Membrane raft	0.000724
	Membrane microdomain	0.000732
	Membrane region	0.000842
MF	Cytokine receptor binding	2.97E-06
	Cytokine receptor activity	1.11E-05
	Immune receptor activity	3.45E-05
	Tumor necrosis factor receptor superfamily binding	4.1E-05
	Death receptor binding	0.000279
	Cytokine binding	0.000851

The KEGG analysis upon the 26 co-DEGs showed that Epstein-Barr virus infection, complement and coagulation cascades and osteoclast differentiation, were mostly enriched terms of co-DEG (Fig. 4B and Table 4). The details of the significantly enriched pathways related to SLE were especially presented in Fig. 4E (https://www.kegg.jp/entry/map05322). The 26 co-DEGs were also mainly enriched in a number of signal pathways, which were highly related to cell proliferation and cell senescence including NF − kappa B signaling pathway, MAPK signaling pathway, Th17 cell differentiation and Toll − like receptor signaling pathway (Fig. 4B\D). To explore the potential effects of co-DEGs upon disease, DO analysis was performed. The results depicted that co-DEGs were mostly enriched in kidney disease, urinary system disease, lymphoblastic leukemia, membranous glomerulonephritis and Human immunodeficiency virus infectious disease. (Fig. 4C and Table 5).

**Table 4:** KEGG analysis of co-DEGs.

	Pathway	*P* value
KEGG	Pertussis	0.000279
	Complement and coagulation cascades	0.000279
	Epstein-Barr virus infection	0.000975
	Staphylococcus aureus infection	0.005291
	Chagas disease	0.005347
	Osteoclast differentiation	0.010595
	Alzheimer disease	0.010841
	Measles	0.010841

**Table 5:** DO analysis of co-DEGs.

	Disease	*P* value
DO	Nephritis	3.54E-06
	Kidney disease	3.87E-06
	Urinary system disease	5.21E-06
	Lymphoblastic leukemia	0.00023
	Head and neck carcinoma	0.000562
	Head and neck cancer	0.000607
	Membranous glomerulonephritis	0.001139
	Alzheimer's disease	0.001362

### Protein–protein interaction network (PPI) network construction

**Figure 5:**
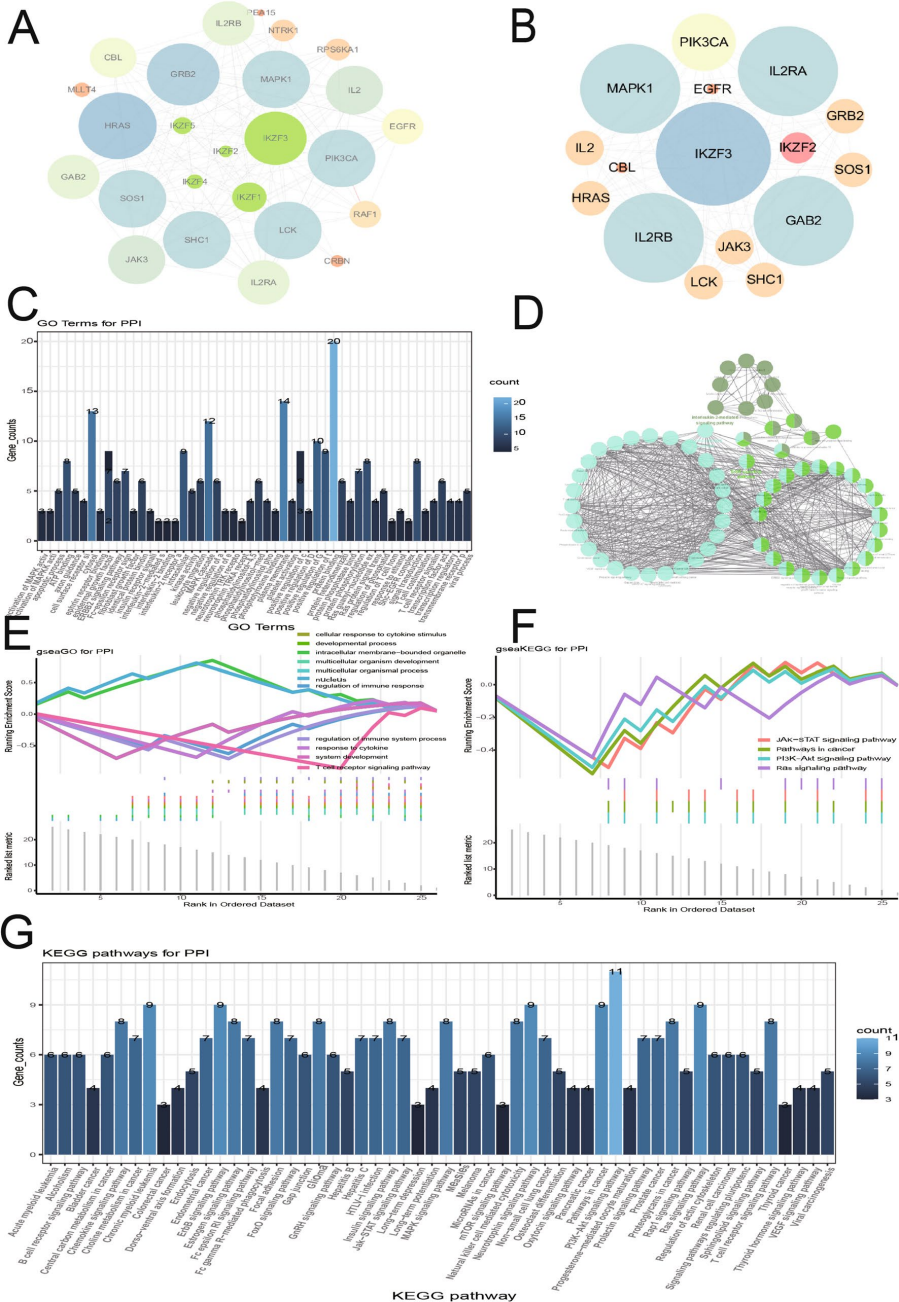
Protein–protein interaction network and its associated functional clusters. (**A**) STRING database was utilized to construct IKZF2 correlated PPI network. (**B**) Cytoscape was applied for visualization of the PPI network. Then, cytoscape plug-in MCODE was used to identify highly interconnected clusters and eventually the functional cluster 1 was obtained. (**C**) DAVID was adopted to annotate GO terms of special functional cluster from PPI network. (**D**) GO terms of the functional cluster 1 was annotated through ClueGO plug-in. (**E**), (**F**) GSEA was performed upon the genes involved in IKZF2 related PPI network. (**G**) pathway enrichment analysis on the genes of functional clusers from PPI network by the means of DAVID. Abbreviation, *PPI* protein–protein interaction network, *GSEA* gene set enrichment analysis, *DAVID* the Database for Annotation, Visualization, and Integrate Discovery.

To identify the possible influence of IKZF2 on the development of LN, PPI network (Fig. 5A, B) was extracted from STRING database, comprising of 25 genes and 144 pairs of interaction. Enrichment analysis was carried out upon these IKZF2-associated genes by DAVID database. The results showed that those were mainly related with MAPK cascade, EGFR signal pathway, ERBB2 signal pathway, actively regulation of GTPase activity, Fc-ε receptor signal pathway, Phosphatidylinositol mediated signal transduction, Leukocyte migration, etc. (Fig. 5C and Table 6). Moreover, categorization by signal pathway revealed that Chronic myeloid leukemia, ErbB signal pathway, Glioma, Neurotrophin signaling pathway, PI3K-Akt signal pathway, Estrogen signal pathway and T cell receptor signal pathway were the hub enriched pathways (Fig. 5G and Table 7).

**Table 6:** GO analysis of PPI.

Go terms	*P* value
MAPK cascade	1.24E-14
Epidermal growth factor receptor signaling pathway	1.01E-10
ERBB2 signaling pathway	1.47E-09
Positive regulation of GTPase activity	2.78E-08
Fc-epsilon receptor signaling pathway	1.13E-07
Phosphatidylinositol-mediated signaling	2.8E-07
Leukocyte migration	5.65E-07
Regulation of phosphatidylinositol 3-kinase signaling	3.57E-06
Protein phosphorylation	2.64E-05
Positive regulation of transcription from RNA polymerase II promoter	2.94E-05
Axon guidance	5.96E-05

**Table 7:** KEGG analysis of PPI.

Pathway	*P* value
Chronic myeloid leukemia	1.16E-12
ErbB signaling pathway	5.59E-12
Glioma	5.17E-11
Neurotrophin signaling pathway	7.77E-11
PI3K-Akt signaling pathway	5.42E-10
Endometrial cancer	1.05E-09
Estrogen signaling pathway	1.06E-09
T cell receptor signaling pathway	1.14E-09
Non-small cell lung cancer	1.66E-09

In order to further verify the latent functional implication of IKZF2 correlated genes in the PPI network, GESA analysis was conducted, and four mostly enriched GO terms were observed, including Cell intima binding organelle, Multicellular biological development, Multicellular biological process and Animal organ development (Fig. 5E). In addition, the genes in PPI network were mainly enriched in PI3K-Akt signal pathway, The cancer related pathway, JAK-STAT signal pathway and Ras signaling pathway (Fig. 5F). Next, we applied cytoscape plugin Molecular Complex Detection (MCODE) to identify highly interplayed clusters in the IKZF2 associated PPI network and finally obtained two functional clusters. And then, we utilized clueGO to annotate functional cluster 1 for its higher score (Fig. 5D). The results showed that multiple genes in functional cluster 1 were mainly enriched in two or three kinds of biological functions and pathways.

### Construction of the ceRNA regulatory network

**Figure 6:**
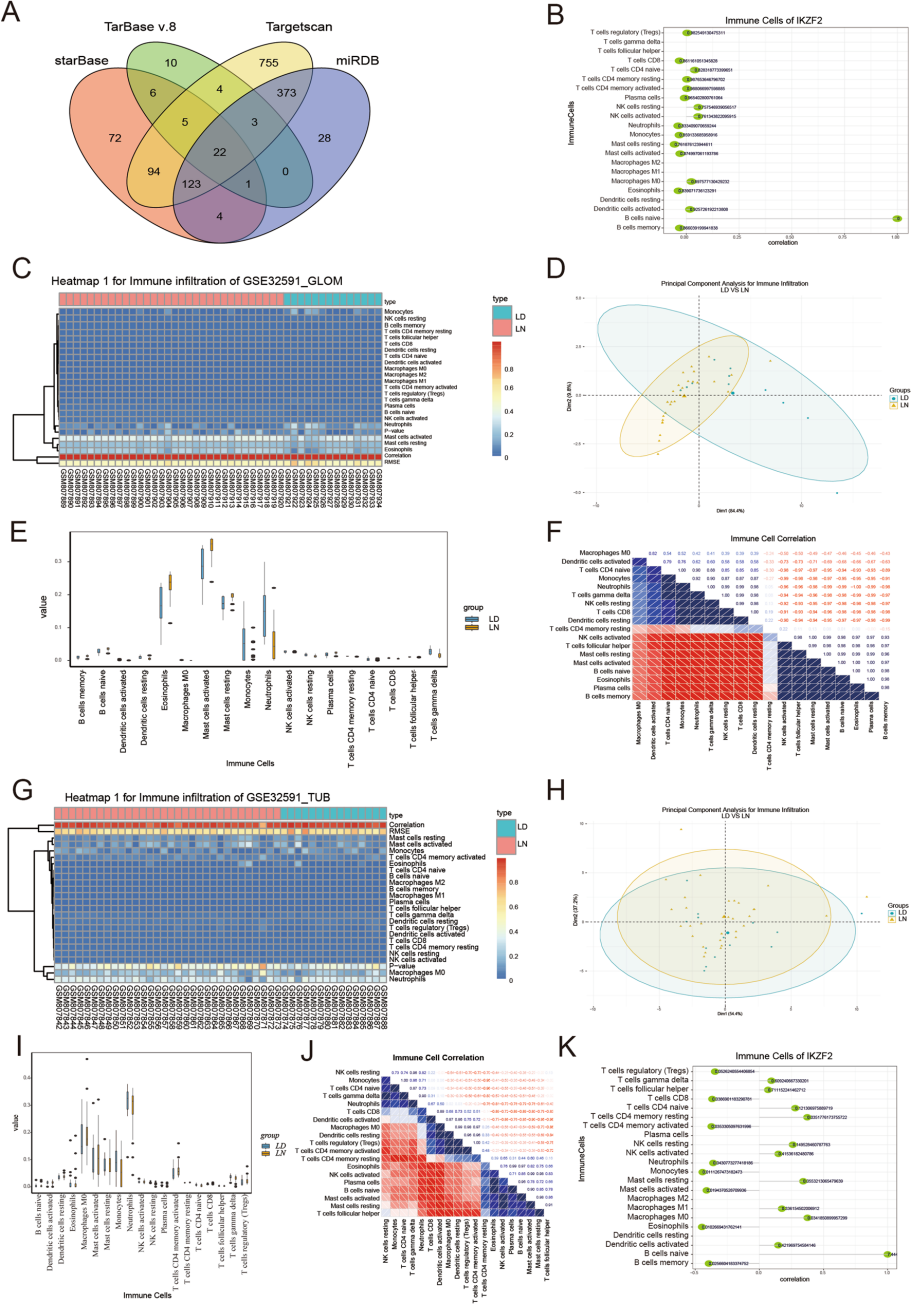
ceRNA regulatory network construction and Immune infiltration analysis. (**A**) The Venn diagram denoting the intersection of the miRNA databases. (**B**) The lollipop figure of correlation between the expression level of IKZF2 and the relative abundance of 22 kinds of immune cells. The abscissa was the correlation score, the ordinate was the immune cells, and the numerical value represented the *p*-value. (**C**) Immune infiltration analysis upon the 26 co-DEGs was performed by the means of CIBERSORT algorithm. The relative abundance of immune infiltration was exhibited in an ascending order from red to blue. The pink strip and the blue-green one represented LN group and normal control respectively. (**D**) The distinction of immunocyte infiltration between LN and normal group based on PCA algorithm. (**E**) Statistics on the distinction of 22 types of immune cell abundance between LN group and normal one. (**F**) The heatmap of correlation between the expression level of IKZF2 and the relative abundance of 22 kinds of immune cells (**G**), (**K**) Immune infiltration abundance of co-DEGs and the correlation between 22 types of immune cells from TUB group of GSE32591 dataset.

**Figure 7:**
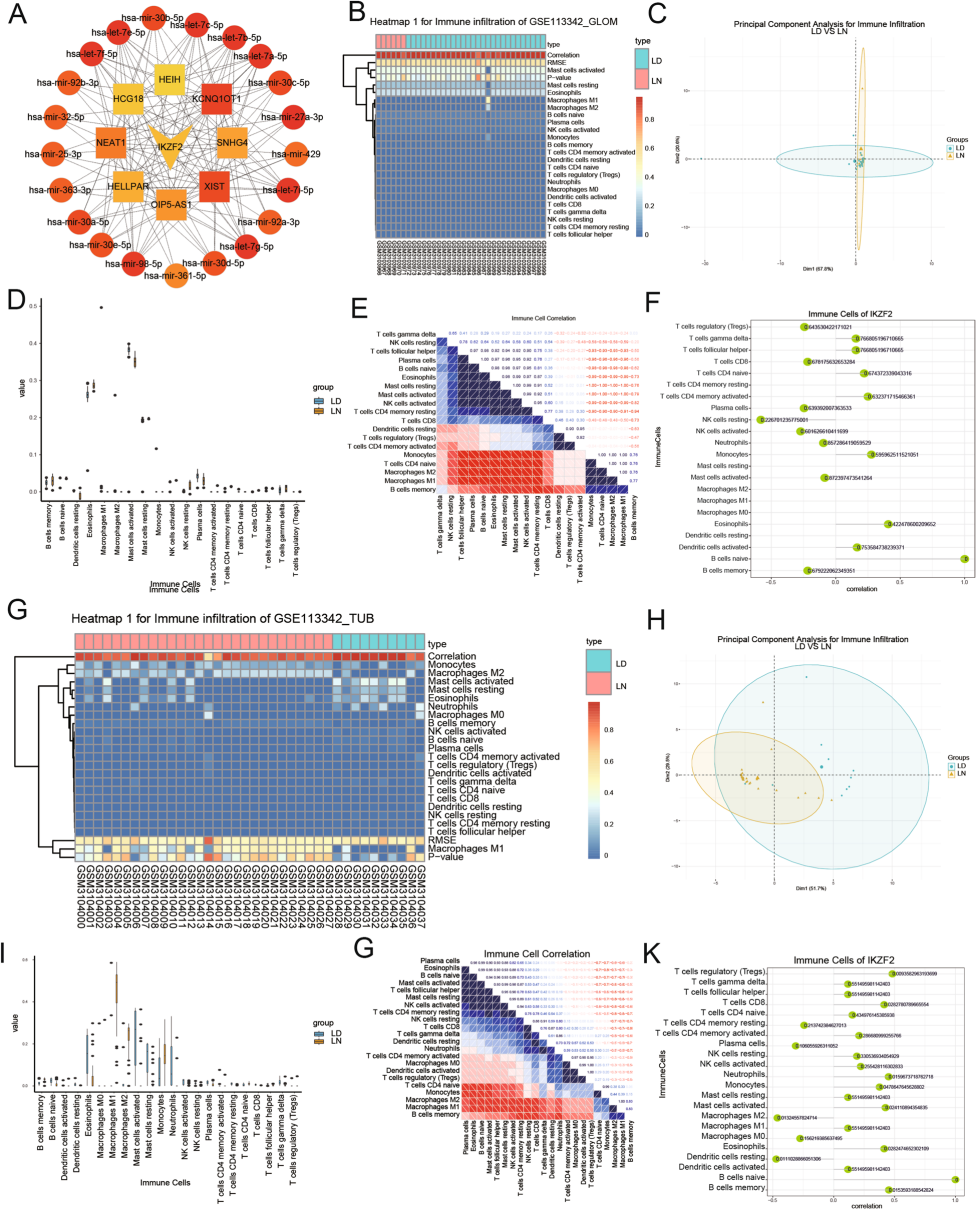
Immune infiltration analysis. (**A**) The cytohub plug-in in cytoscape was utilized for visualizing ceRNA regulatory network including lncRNA-miRNA-mRNA interaction information. (**B**)–(**E**) Immune infiltration abundance of co-DEGs and the correlation between 22 types of immune cells from GLOM group of GSE11342 dataset. (**F**)–(**K**) Immune infiltration abundance of co-DEGs and the correlation between 22 types of immune cells from TUB group of GSE11342 dataset.

We next set out to better comprehend the potential regulation mechanism of IKZF2 in the progression of LN by establishing IKZF2 related ceRNA regulatory network. First, we utilized four databases to predict miRNA, which targeted the gene expression of IKZF2 and eventually obtained 22 unique miRNAs in all databases (Fig. 6A). Subsequently, 1178 LncRNA interplaying with the above 22 miRNAs were forecast based on miRNet database. And finally, cytoscape software was utilized for visualizing ceRNA regulatory network, which was composed of lncRNA-miRNA-mRNA interaction information. The cytohub^48^ plug-in in cytoscape was used to identify the key lncRNA-miRNA-mRNA regulatory mechanism in the ceRNA network (Fig. 7A and Supplementary1_miRNA.xls).

### Immune infiltration analysis

We further analyzed the relative abundance of immune infiltration of co-DEGs in 22 immune cells types from LN and normal tissues. The results exhibited that there was a significant distinction between LN samples and LD in the GLOM group from GSE32591 dataset (Fig. 6C). PCA analysis also confirmed this difference of immune cell infiltration between LN samples and LD (Fig. 6D). Subsequently, we conducted statistical analysis upon the 22 immune cells types to determine their difference in the two groups. Statistical analysis revealed that the significant difference of abundance in the 22 immune cells types were mainly focus on the following immune cells: neutrophils, monocytes, eosinophils and macrophages M0 (Fig. 6B). Moreover, Spearman correlation was calculated to reveal the link between 22 types of immune cells in LN tissues. The results indicated four immune cell subsets, including naive T cells, monocytes, neutrophils, and γδT cells, were significantly correlated (Fig. 6E). The results also revealed the significant correlation between IKZF2 and naïve B cell, NK cell activation, NK cell rest and other immune cells. (Fig. 6F).

In addition, the immune infiltration between LN/LD samples from other group of datasets, including TUB group in the dataset GSE32591 (Fig. 6G-K), GLOM group (Fig. 7B–F) and TUB group (Fig. 7G–K) in the dataset GSE113342 was also analyzed in our study. Overall, in TUB group between LN/LD samples, the significant difference of abundance in the 22 immune cells types were mainly focus on the following immune cells (Cor > 0.5): γδT cells, T follicular helper, M1 Macrophage, Mast cell resting and Dendritic cell activated. In GLOM group between LN/LD samples, the results obviously showed the significant correlation between IKZF2 and kinds of T cell, NK cell activated, neutrophils, and other immune cells (Figs. 6, 7).

### Clinical characteristics correlation analysis and relative risk assessment

**Figure 8:**
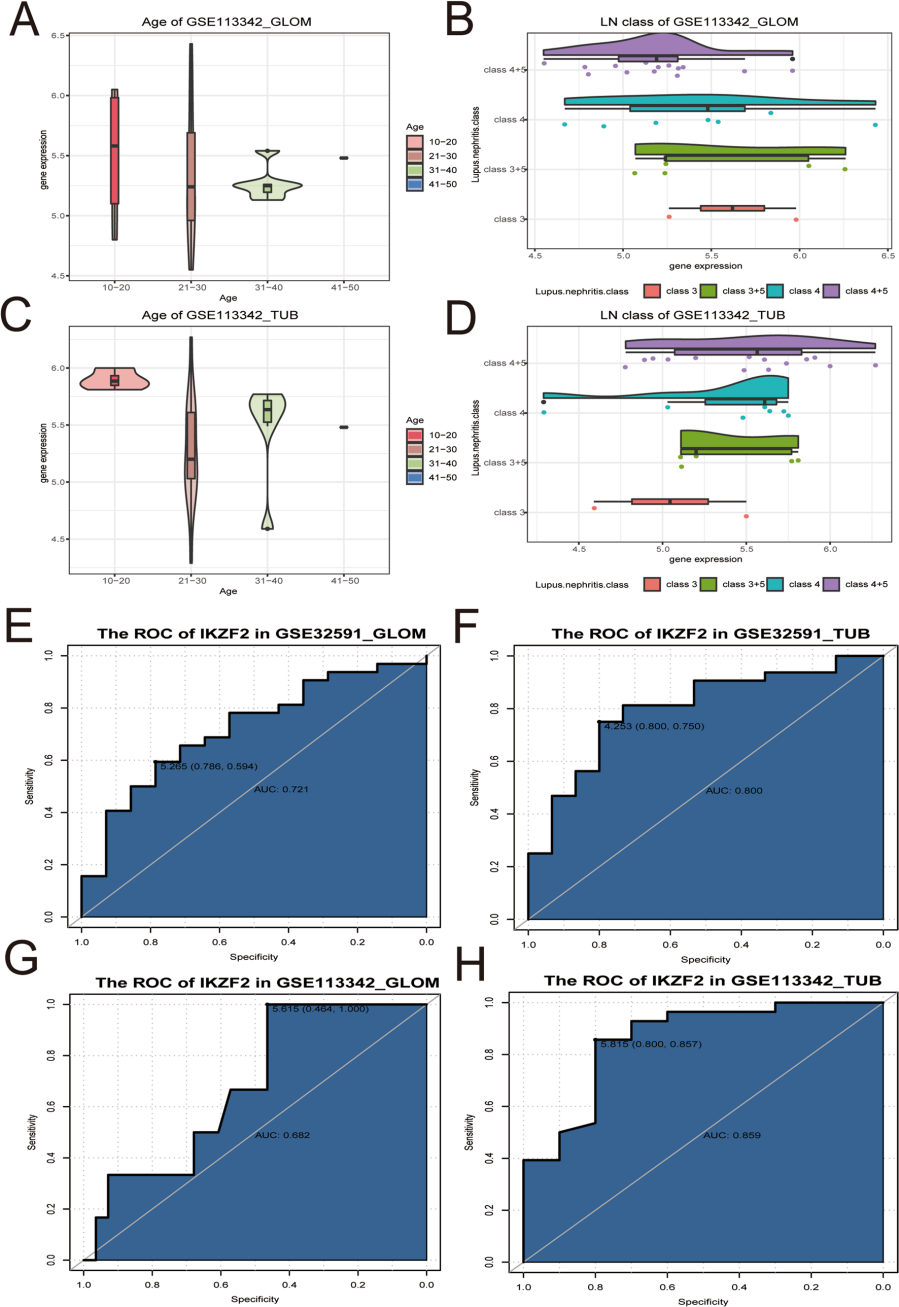
Clinical characteristics correlation analysis and relative risk assessment. (**A**\**C**) Correlation analysis between the expression level of IKZF2 and the age of LN patients. The x-axis represented the age of LN patients, and y-axis denoted the expression of IKZF2 in the GLOM and TUB group from GES11342 dataset. Different color represented distinct group of age. (**B**\**D**) Correlation analysis between the expression level of IKZF2 and the lupus nephritis class. The x-axis represented the expression of IKZF2 in the GLOM and TUB group from GES11342 dataset, and y-axis represented the disease grade of lupus nephritis, which included class3, class3 + 5, class4 and class4 + 5. (**E**)–(**H**) ROC curve of IKZF2 expression level to distinguish LN samples and LD in the four sets of data. The results showed that IKZF2 revealed a good AUC value in GSE32591_GLOM, GSE32591_TUB and GSE113342_TUB datasets.

In order to verify the clinical significance of IKZF2, we preformed correlation analysis between the gene expression level of IKZF2 and the age of LN patients and related LN class. Although, no significant correlation was found between the IKZF2 expression level and the age of relative patients (Fig. 8A\C), the results exhibited that the IKZF2 expression was related with the disease grade of LN.

In the GLOM group, higher LN class revealed higher expression of IKZF2. In the TUB group, on the contrary, higher disease grade of LN denoted lower expression of IKZF2. (Fig. 8B\D) After that, we next explored whether IKZF2 could distinguish LN samples and LD. The areas under the ROC curves were 0.721, 0.80, 0.682, and 0.859 for IKZF2 in GSE32591_GLOM, GSE32591_TUB,GSE113342_GLOM, and GSE113342_TUB datasets, which implicated that IKZF2 might be a potential biomarker for distinguishing LN patients and healthy ones. (Fig. 8E–H).

### Validation with other GEO datasets

**Figure 9:**
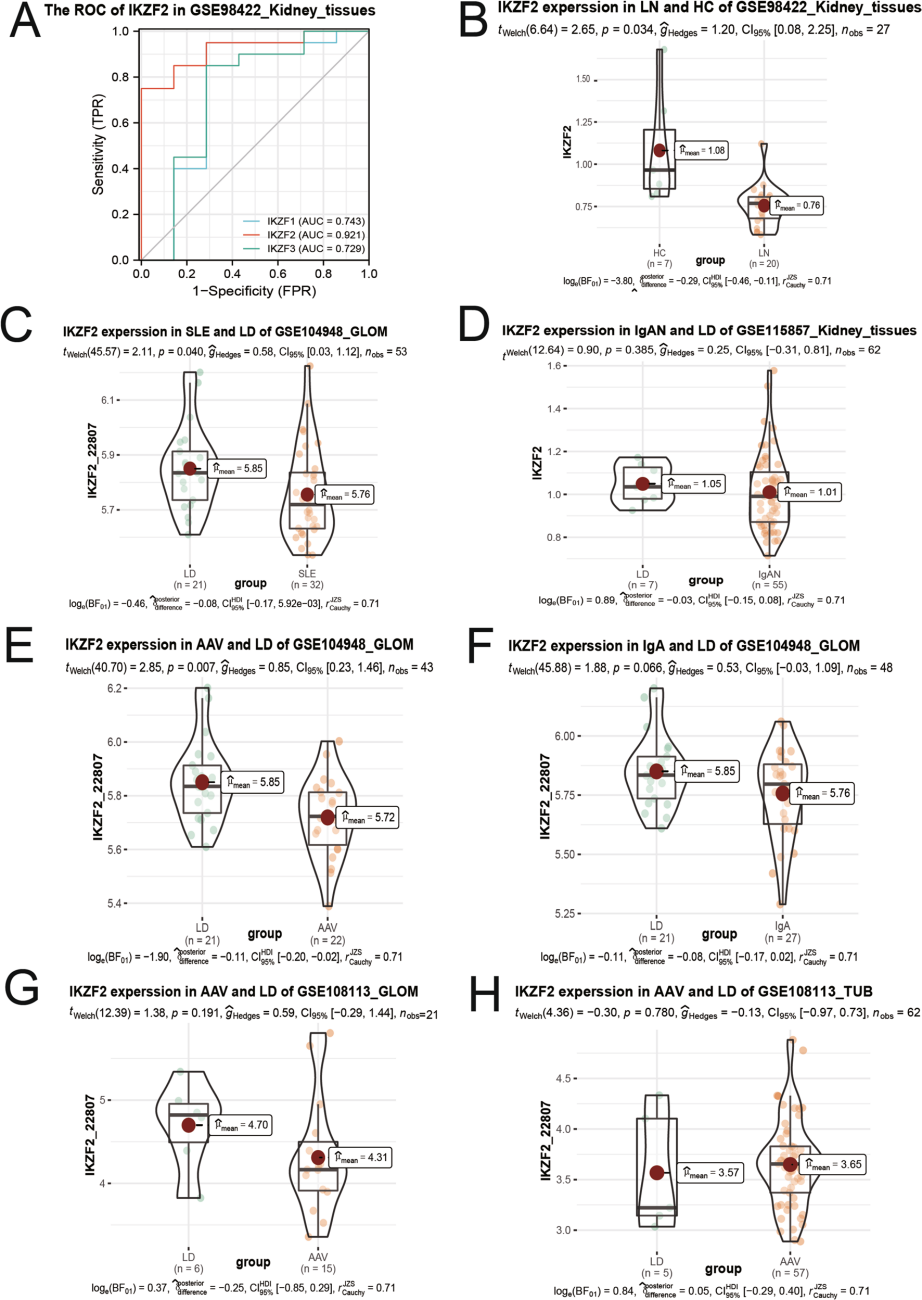
Validation with other GEO datasets. (**A**) ROC curve of IKZF family expression level to distinguish LN samples and LD in the dataset of GSE98422. (**B**\**C**) Comparison of IKZF2 expression value between LN/LD samples by datasets of GSE98422 and GSE104948. (**D**\**F**) Comparison of IKZF2 expression value between IgA nephropathy and normal by datasets of GSE104948 and GSE115857. (**E**\**G**\**H**) Comparison of IKZF2 expression value between ANCA associated vasculitis and normal by datasets of GSE104948 and GSE108113.

To verify our conclusion in a broader range, we analyzed the differentially expressed level of IKZF2 between LN and normal by datasets of GSE98422^49^ and GSE104948^50^ (Fig. 9A–C). The areas under the ROC curves were 0.921 for IKZF2 in GSE98422. And all datasets showed that IKZF2 was consistently downregulated in LN, illustrating a satisfactory reliability of our research.

Moreover, we valued IKZF2 expression between IgA Nephropathy and normal by datasets of GSE104948^50^ and GSE115857 (Fig. 9D\F), and IKZF2 didn’t exhibit significant transcriptional differences in the two datasets. We also examined the differentially expressed level of IKZF2 between ANCA associated vasculitis and normal by datasets of GSE104948^50^ and GSE108113^50^ (Fig. 9E\G\H). IKZF2 was downregulated in ANCA associated vasculitis of GSE104948, but no significant difference was found in GSE108113.

## Discussion

SLE is a chronic autoimmune disease characterized by loss of immune tolerance and various organ or tissue damage. About 25–50% patients diagnosed with SLE have symptoms of LN at onset, and approximately 60% of adult patients with SLE develop renal signs or symptoms during the disease course. LN remains/ed a major cause of morbidity and death for SLE patients^51^. Laboratory test has made great strides in diagnosis and therapy, but the incidence of renal flares ranged from 27 to 66%^52^. However, SLE is a multifactorial process with potential involvement of different organs, and varied in its clinical severity, and the pathogenesis remains unclear. Hence, more accurate prediction and evaluation remain the great challenges for the improvement of LN outcomes.

The tissue injury or clinical manifestations are influenced by genetic, epigenetic, environmental, hormonal, and immunoregulatory factors^2^. Many genes which encode related proteins have been found potential candidates associated with SLE, such as DNase I, DNase­γ, DNase III and TREX1 involved in DNA clearance, C1q and C4 od the complement pathway and other functions (ACP5, AGS5, FASL and STING)^51^ and Fc receptors^53^. More than 80 genes have been identified associated with SLE by GWAS. Multiple signaling pathways were involved including type I interferon production (IRF5, IRF7, TLR7, TLR8, TLR9 and STAT4)^54^, nuclear factor-kappa B (NF-κB) signaling pathway^55^ and effector and regulatory CD4( +) T cells especially Th 17 cells^56^. People has focused on the possibility that viruses may trigger SLE for decades. It suggested that CD8 + T cells are unable to control Epstein–Barr virus (EBV)-infected B cells in SLE patients, because of the higher viral load and faster seroconversion to infection and the molecular similarity between EBV nuclear antigen 1 and the common lupus autoantigen Ro^2,57–59^.

In the current study, gene expression analysis was applied to identify co-DEGs and possible predictor factors between LN patients with healthy controls. A total of 26 co-DEGs, 717 miRNA and 10,125 lncRNA were identified and the PPI network was conducted. Most of the variable gene and pathways involved have been previously described in previous researches. Based on the analysis of co-DEGs expression, the IKZF2 was found to be down-regulated in the GLOM group and TUB group of the disease group. In order to explore the potential function of IKZF2 in LN patients, we analyzed the abundance of co-DEGs in 22 types of immune cells in LN tissues and normal tissues. The IKZF2 was correlated with immune cells including initial B cells and NK cell. The result showed the excellent value of IKZF2 for the AUC and we can substantially distinguish LN and healthy controls tissues.

The IKZF2 gene encodes a member of the Ikaros family of zinc-finger proteins including Ikaros, Aiolos and HELIOS, which are hematopoietic-specific transcription factors involved in the regulation of lymphocyte development^60^. IKZF2, also known as HELIOS, a transcription factor expressing in 60–70% Foxp3þ Treg cells, recently has been demonstrated that it played a role in controlling certain aspects of Treg-suppressive function, differentiation, and survival^61^. Treg cells play an important role in maintaining immune self-tolerance by suppressing effector T and B cells. And it’s believed that abnormalities of Treg cells may lead to T and B cell hyperactivity and contribute to the pathogenesis of SLE. It’s reported that Tregs expressing IKZF2 have stronger immunosuppressive capacity^62^. In vitro experiments, it was found that IKZF2 deficient mice would acquire an auto-inflammatory phenotype similar to rheumatoid arthritis. And activated CD4 + and CD8 + T-cells, T-follicular helper cells and germinal center B-cells were noticed increased leading to production of autoantibody in circulation^63^. In recent study, CD4 + CD25 − Foxp3 + T cells major expressing IKZF2 were indicated significantly increased in active SLE patients. Detailed cohort analysis revealed increased proportions of CD4 + CD25 − Foxp3 + T cells in SLE patients especially LN patients. And the same type T cell was detected in urine sediment samples of patients with active glomerulonephritis and correlated with the extent of proteinuria^64^. Related data suggested that association between the percentage of FoxP3 + IKZF2 + Treg cells and the SLEDAI score is positive. There is a higher percentage of FoxP3 + IKZF2 + Treg cells in patients with more active disease compared to the patients with inactive disease or healthy donors^62^. Thus, it’s assumed that CD4 + CD25 − Foxp3 + T cells might serve as an important tool to recognize and monitor SLE patients with renal involvement. However, IKZF2 is considered as a marker of T cell activation and proliferation^62,65^. It’s possible that IKZF2 might become a new index for prediction and evaluation of LN.

Our study is the first study to explore the molecular mechanisms of IKZF2 in LN by multiple datasets and applying the immune infiltration analysis. Moreover, the AUC of IKZF2 expression had very high diagnostic value in each dataset based on ROC curves, revealing it might be a potential biomarker for distinguishing LN patients and healthy ones. However, due to the ethical and traumatic reasons, SLE patients without nephritis may not be punctured by the kidney. We cannot compare patients with nephritis against patients without nephritis. Nevertheless, we tried our best to obtain all available data and also analyzed clinical characteristics correlation and relative risk assessment. In addition, we valued IKZF2 expression in IgA Nephropathy and ANCA associated vasculitis, verifying our conclusion in a broader range. Besides, further experimental verifications are necessary to illuminate the biological functions of IKZF2 in LN. Subsequently, we would like to perform more careful examinations of the function of IKZF2 in LN by combining in vivo and in vitro experiments. Therefore, we consider that our study may help to investigate the progress of LN, and that IKZF2 may become biomarkers and therapeutic targets of LN in the future.

## Conclusion

The absence of our patient cohort makes it difficult to verify the results. However, previous researches focused on the expression of IKZF2 in peripheral blood to evaluate the prognosis of the disease, and our study provided some evidence and new ideas about new sights for prediction and evaluation of LN. Our bioinformatics analysis identified the gene IKZF2 may be critical in the prediction and evaluation of disease activity. We hope this study may provide some potential evidence and ideas for the future treatment and monitoring of LN from new insights.

## Supplementary Information

Supplementary Information.

## Abbreviations

LN: Lupus nephritis
co-DEGs: Common differentially expressed genes
ROC: Receiver operating characteristic
SLE: Systemic lupus erythematosus
ESKD: End-stage kidney disease
GWASs: Genome-wide association studies
PBCs: Peripheral blood cells
PPI: Protein–protein interaction network
GEO: Gene Expression Omnibus
TUB: Tubulointerstitium
GLOM: Glomerulus
DEGs: Differentially expressed genes
GO: Gene Ontology
KEGG: Kyoto Encyclopedia of Genes and Genomes
DO: Disease Ontology
MCODE: Molecular Complex Detection
DAVID: Database for Annotation, Visualization, and Integrate Discovery
GSEA: Gene Set Enrichment Analysis
ceRNA: Competing endogenous RNA
PCA: Principal components analysis
AUC: Area under the ROC curve
MCODE: Molecular Complex Detection
NF-kB: Nuclear factor-kappa B
EBV: Epstein–Barr virus
